# Profile: Morgan David Enoch

**DOI:** 10.1192/pb.bp.114.048066

**Published:** 2015-06

**Authors:** Roxanne Keynejad

A benchmark of fame in the modern world, a quick Google search for Dr Morgan David Enoch brings up an intriguing set of results. These range from a BBC article, ‘Spotting the Royal stalkers’,^1^ a *BMJ* case report on ‘Exorcism for schizophrenia’^2^ and Ian McEwan’s *Enduring Love*,^3^ to Mersey Regional Health Authority’s implementation of care in the community and books, including *I Want a Christian Psychiatrist*^4^ and *Uncommon Psychiatric Syndromes*:^5^ now out of print, the paperback fourth edition is currently available from online sellers, second-hand, for over £1000.

The spectrum of today’s search results for this 88-year-old emeritus consultant psychiatrist of Royal Liverpool University Hospital, fellow of the Royal College of Psychiatrists, member of its Council for over 20 years and Court of Electors for 8, due shortly to publish his autobiography, offers just a glimpse of the diverse tales Dr Enoch might relate, should you be lucky enough to meet him.

## Waziristan

With a surname meaning ‘he who walks with God’, Morgan David Enoch was a sixth-form student due to read theology at the end of World War II. David’s mother, who worried about his sister attending a hairdressing course in Swansea, 8 miles from the family home, was shocked when, after a letter from the King of England, he was soon serving with the British army in India’s north-west frontier, ‘facing the forces of the Faqir of Ipi at the very place where fighting has been heaviest for the [past] 10 years’, aged 18. Later, following a War Office Selection Board, he trained at the Indian Military Academy, ‘the Sandhurst of India’ at Dehra Dun, was commissioned into the Royal Artillery and posted to the second Indian (Sikhs) Field Regiment in 1947.

During this time, he says ‘while angry with God for obstructing my chosen path, I had four exciting years in India. I heard Jawaharlal Nehru and Mohammed Ali Jinnah address vast crowds: I witnessed the birth of two great nations and the process of India’s violent partition’. Playing rugby for the British Army in India, David returned to Woolwich Arsenal on leave, expecting to continue playing but was indignant to be ordered back to India to complete his outstanding months’ National Service. He says the physical and mental suffering he observed during his army career is what called him to medicine. After eventual military discharge, he ‘went around the London teaching hospitals and liked St Thomas’ Medical School. I think the uniform helped at interview’.

## Medical training

Qualifying in 1954, following house jobs Dr Enoch returned to Wales to work at St David’s Mental Hospital, Camarthen, as his father, ‘a hewer of coal in 2 feet 9 inches’ was dying of silicosis. Of his first years in psychiatry, he recalls the routine use of the hypnotic paraldehyde at night, prescribing ‘quite effective’ Drinamyl (amobarbital and dextroamphetamine, later known as ‘purple hearts’), deep insulin coma therapy and, for the first time, an antipsychotic: chlorpromazine.

Following his father’s death in 1958, he returned to London, then ‘the supreme place to train’, obtaining a medical registrar post at University College Hospital, working for Professor Baron Max Leonard Rosenheim, Dr Roger Tredgold and Dr Desmond Pond, later president of the Royal College of Psychiatrists. In the mid-fifties, after the Institute of Psychiatry course and obtaining his Diploma in Psychological Medicine, Dr Enoch witnessed the introduction of antidepressants, beginning with imipramine and amitriptyline. He later obtained a senior registrar post divided between the London Hospital (now the Royal London Hospital) and Runwell Mental Hospital, Essex, which opened after World War II in response to shell shock – the last mental hospital built in Britain.^6^

Dr Enoch recalls Runwell’s physician superintendent, Dr Rolf Ström-Olsen, as ‘one of the best physicians I ever met. He was Norwegian, abrupt and highly intelligent’. He recollects Runwell’s units divided into neurosis and psychosis, conferences clouded in cigarette smoke, treating patients for the first time with out-patient electroconvulsive therapy and the introduction of newer antipsychotics, like haloperidol. This dynamic clinical, educational and research environment was to prove instrumental in inspiring his future writing, yielding a lifelong dedication to evidence-based improvement in clinical practice.

### Uncommon Psychiatric Syndromes

Dr Enoch recalls presenting a patient at Runwell Hospital, whose complaint was that ‘my wife’s an impostor’. Phrases began to occur to him describing the psychodynamic processes at work: ‘His wife was ”the fallen idol”, hence the delusion of doubles. How do you resolve loving and hating someone at the same time? By splitting. Is apperception not a wonderful word? To perceive with feeling. That is the essence of Capgras psychopathology.’

After further research, Dr Enoch’s paper on Capgras syndrome won the bronze medal and prize of the Royal Medico-Psychological Association in 1962 and was published in *Acta Psychiatrica Scandinavica* in 1963,^7^ the same year he won the Gaskell Prize. He developed an increasing interest in rare presentations, from De Clérambault’s syndrome, to folie à deux, Tourette’s and more, which yielded his first book. The first edition of *Some Uncommon Psychiatric Syndromes*, a bestseller, was published in 1967, with further editions in 1979, 1991 and 2001. Dr John Pollitt, President of the Section of Psychiatry of the Royal Society of Medicine, predicted that it ‘well deserves to be placed as a classic’;^8^ it has been translated into French, German, Turkish and Japanese.

Dr Enoch expected his ‘uncommon syndromes’ to remain rare and was surprised by their later *cause célèbre* status. ‘I never thought, for example, that patients with Munchausen syndrome by proxy would become so important. They became huge’. The diagnosis was questioned publicly, after several child killing convictions were overturned, including that of lawyer Sally Clark. In these cases, the diagnosis was used inappropriately for paediatric assessment, without involvement of a psychiatrist. By contrast, high-profile cases incorporating psychiatrist assessment, such as that of nurse Beverley Allitt, now detained at Rampton Secure Hospital, demonstrated the contemporary relevance of the diagnosis.

To this day, Dr Enoch receives telephone calls from readers of *Uncommon Psychiatric Syndromes* from around the world; he remains fascinated by delusional disorders. ‘I’d like to know far more about emotions. How can feelings affect thought? Psychodynamics remain all important. This book has been lived’. Although he never met Ian McEwan, who referenced the text in *Enduring Love*, Dr Enoch ‘approved’ of the novel, unlike the film *Fatal Attraction* (1987, director A. Lyne), which did not depict true De Clérambault’s syndrome, because ‘the protagonists had an initial affair: usually there is no contact’.

After the success of *Uncommon Psychiatric Syndromes*, Dr Enoch wrote other well-received books, including *Healing the Hurt Mind: Christian Faith and Psychiatry*.^9^ Now in its 11th edition, its title was inspired by a patient’s letter, kept to this day, stating that ‘Dr Enoch was the first person who listened to me and asked me how I am. Healing began for me today’. In the most recent, *I Want a Christian Psychiatrist*,^4^ he returns to his theological roots. ‘You don’t necessarily need a Christian psychiatrist: you need a competent psychiatrist who must respect your faith’.

## Clinical career

Dr Enoch recalls obtaining his first consultant post at Shelton Hospital, Shrewsbury, after debating Ganser syndrome with Professor Sir William Trethowan, foundation chair of psychiatry at the University of Birmingham, at interview. He would later invite Trethowan to contribute to the chapter on Ganser syndrome and Dr John C. Barker that on Munchausen syndrome, in the first edition of *Uncommon Psychiatric Syndromes*.

It was at Shelton Hospital that Dr Enoch introduced regular teaching, therapeutic communities and care in the community. He recalls meeting Enoch Powell, then expounding de-institutionalisation as minister of health. He worked with Barbara Robb on a national campaign for elderly care in hospitals, contributing a chapter to her book, *Sans Everything: A Case to Answer*.^10^ He remembers ‘feeling encouraged’ at the time by the *Sunday Times*’ article series on care in the community by Marjorie Wallace, who later founded the mental health charity SANE, with telling photographs by Lord Snowden.

Dr Enoch was later head-hunted to take on the new post of consultant psychiatrist and senior clinical lecturer at the new Royal Liverpool University Hospital’s ‘superb’ department of psychiatry, later including beds at Rainhill Mental Hospital. He is still remembered as a dynamic and enthusiastic teacher, as evidenced by Dr Gamal Hammad’s description of him as ‘a charismatic guru, a wonderful mentor and a visionary’.^11^

**Figure:**
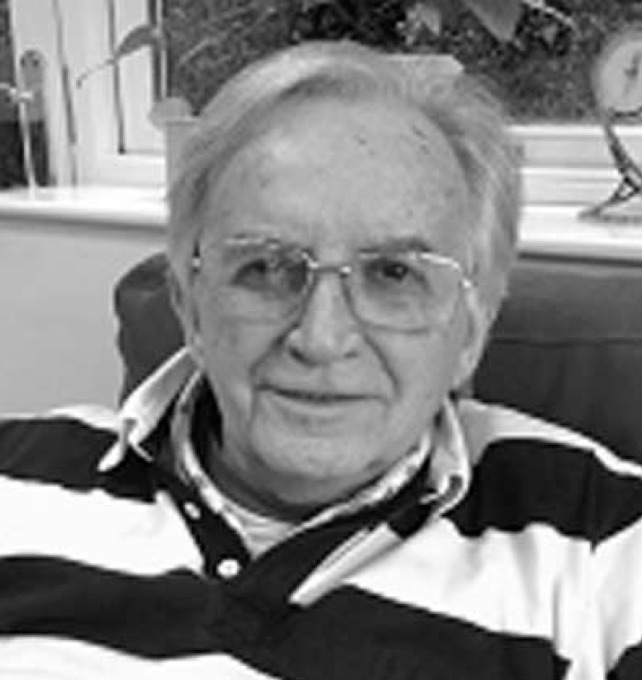
Dr Enoch at his home in Cardiff, March 2014.

He always enjoyed encouraging the next generation of psychiatrists and made a point of involving students and nurses in ward rounds: ‘I wanted the doctors and students to see the full range of psychiatric disorders. I used to remind them, ”Rare things rarely occur”.’ At the opening of Shelton Hospital’s replacement, the Redwoods Centre, in 2011, his second wife, Anne, a retired headmistress, recalls him being treated ‘like a celebrity... they’d all read his book’.

Above all, Dr Enoch strove to teach and practise psychiatry as a holistic discipline, examining each and every patient: ‘I liked psychiatry because it deals with the whole person: their body, mind and spirit. I wanted to do something exciting while staying true to my faith. A good psychiatrist is prepared to listen, know their stuff and gather it all together into a diagnostic formulation. It doesn’t mean you have all the answers, but the diagnosis is the first step to management and treatment.’

## On psychiatry

Dr Enoch is a staunch supporter and advocate of psychiatry as a compelling specialty with much still to be discovered: ‘It’s a new frontier of medicine... We know so little about the human brain: the heart is a pump, but you love with the hypothalamus. We thought that scans would give us clear-cut answers but they have not. What is consciousness? I have thirty books on it but no one can say where, how, what. It is remarkable what can arise from the unconscious. I thought that fMRI would locate jealousy, De Clérembault, because those syndromes are so specific: monomanias with one delusion. The fact that we cannot locate them suggests that the brain’s connections hold the answers. But can this brain really have the capacity to understand itself?’

The specialty remains compelling for Dr Enoch, even after more than 50 years; he continues to advocate keeping psychiatric wards within medical hospitals: ‘Psychiatry is the most intriguing of all specialties: you deal with the whole

**Figure:**
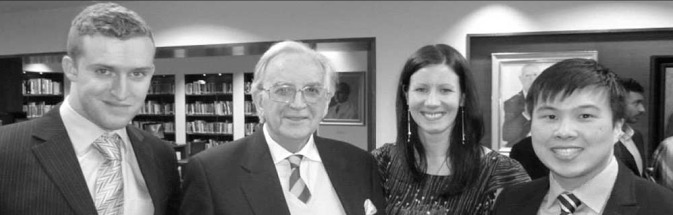
Meeting the Royal College of Psychiatrists’ Pathfinder Fellows, January 2014.

person. You must be a first-class physician: I have picked up lung cancer, brain tumours, pernicious anaemia, cardiac lesions, thyrotoxicosis. We are physicians of psychological medicine. Recalcitrant cases not medically understood are referred to us. You listen and discover things other doctors miss.

Patients come to you broken psychologically and emotionally, intent on suicide, and get better. Is there anything more helpful in society than to heal pain: physical and psychological? Psychiatric illness is an illness like any other: treatable and curable despite what even doctors may think. You need maturity to choose something so difficult, though. You will need hope, positivity and graciousness.’

## On reflection

Having seen his last patient on 31 December 2012 and survived a coronary artery occlusion of ‘99%’, Dr Enoch remains active, lecturing at Cardiff University Medical School until recently. In addition to his forthcoming autobiography, a book of Welsh essays is due for publication in 2014, with several exploring the relevance of the Ten Commandments today. Although he never did complete that theology degree, he has continued to preach from age 16 until today, in between spending time with his wife, son and four grandchildren. Considering his rather uncommon life, he reflects that: ‘I have been greatly blessed. I am very grateful for a very exciting life; it’s still exciting. I have enjoyed psychiatry’s riches in helping people in great depth.’

When we last met, Dr Enoch was looking forward to meeting recipients of the Royal College of Psychiatrists’ Pathfinder Fellowships, considering the next generation with excitement – and a little envy: ‘this is going to be the century of the brain’. Above all, it is the pursuit of learning which he most fervently advocates for us all: ‘I spent 65 years in the game, to realise how little I know. I would love to be starting again, with the knowledge I have now’. His wife adds, ‘He is as enthusiastic today as when he was twenty’. All taught by Dr Enoch, exposed to this enthusiasm, may count themselves truly fortunate. His last piece of advice? ‘Gather your materials from everywhere, but be your own architect.’
